# An exploratory study on the contribution of graduate entry students personality to the diversity of medical student populations

**DOI:** 10.1007/s40037-014-0150-z

**Published:** 2014-11-20

**Authors:** Pedro Marvão, Isabel Neto, Miguel Castelo-Branco, José Ponte, Miguel Portela, Patrício Costa, Manuel João Costa

**Affiliations:** 1 Department of Biomedical Sciences and Medicine, University of Algarve, 8005-139 Faro, Portugal; 2 Faculty of Health Sciences, University of Beira Interior, Covilhã, Portugal; 3 School of Economics and Management, University of Minho, Braga, Portugal; 4 Life and Health Sciences Research Institute (ICVS), School of Health Sciences, University of Minho, Braga, Portugal; 5 ICVS/3B’s, PT Government Associate Laboratory, Braga, Guimarães, Portugal

**Keywords:** Personality, NEO-FFI, Undergraduate medical students, Graduate entry

## Abstract

Studies conducted in medical education show that personality influences undergraduate medical students academic and clinical performances and also their career interests. Our aims with this exploratory study were: to assess the contribution of graduate entry students to the diversity of personality in medical student populations; to assess whether eventual differences may be explained by programme structure or student age and sex. We performed a cross-sectional study underpinned by the five-factor model of personality, with students attending three medical schools in Portugal. The five personality dimensions were assessed with the Portuguese version of the NEO-Five Factor Inventory. MANOVA and MANCOVA analyses were performed to clarify the contributions of school, programme structure, age and sex. Student personality dimensions were significantly different between the three medical schools [*F*_*(10,1026)*_ = 3.159, *p* < .001, $\upeta_{\text{p}}^{ 2}$ = 0.03, π = 0.987]. However, taking sex and age into account the differences became non-significant. There were institutional differences in personality dimensions. However, those were primarily accounted for by sex and age effects and not by the medical school attended. Diversifying age and sex of the admitted students will diversify the personality of the medical student population.

## Introduction

Recent recommendations by professional bodies involved in the evaluation and accreditation of undergraduate medical education are emphasizing the importance of diversifying the population of medical students [1, 2]. Graduate-entry programmes are expected to contribute to such diversification [3–5] as these programmes are expected to admit an alternate pool of medical students who are more similar to the general population, and to ‘widen the access’ to tertiary students who are able to achieve higher learning and academic performance in medical school than secondary students [6, 7].

Historically, in Europe, medical schools admitted by and large 17- to 18-year-old high school leavers to 6–7 year programmes. More recently, government initiatives in some European countries—for example, UK, Ireland and Portugal—have allocated a significant number of medical school places, into identical or shorter (typically 4-year) programmes, to students who have completed tertiary degrees: the ‘graduate entry’ students [3, 4, 8].

In Portugal, the specific availability of places for graduate applicants began in 2007 and is currently 15 % of the current annual medical student intake. There are now eight medical schools, one of which accepts graduate students exclusively. The remaining seven schools accept students through two pathways: the specific pathway for graduate entrants (15 % places) and the national general application and admissions process (residual) which accepts applicants based on ranking of high school academic performance. Therefore, Portugal offers an interesting context to clarify how graduate entry programmes are contributing to the diversity of undergraduate medical student populations.

Personality is an important variable in the context of medical education as it influences such disparate phenomena as academic and postgraduate performances [9, 10], student empathy [11, 12], clinical skills [13, 14], engagement in extracurricular activities [15] and career preferences [16]. The characterization and eventual contribution of graduate entry students’ personality profiles to the diversity of medical student populations across institutions remains mostly unaddressed [10]. The exception is a recent multi-institutional study in Australia, which compared undergraduate and graduate entry schools and found significant but small magnitude differences in conscientiousness and agreeableness between schools [17]. This study was conducted to clarify whether high school and graduate entry students differ in their personality profiles. To this purpose we have chosen another cultural context—Portugal—but have used the same model of personality—the Big Five. The study addressed the following questions related to personality differences between graduate entry and high school entry students: First, do graduate entry students contribute to diversify the personality profiles of medical student populations across institutions? Second, do graduate entry students contribute desirable personality dimensions in a doctor such as higher conscientiousness and openness to experience and lower neuroticism? Third, can diversity in personality across schools be explained by the sex and age of the students? The three medical schools analyzed offer different programme types: University of Beira Interior and University of Minho run 6-year programmes accessed through the general admissions process; University of Minho also runs a 4-year graduate entry parallel track programme and University of Algarve runs a 4-year graduate entry only programme. These medical schools are also located in different regions of Portugal; University of Minho is in the north, University of Beira Interior is in the centre and University of Algarve in the south. Therefore, these medical schools could provide interesting contexts for the present study.

## Materials and methods

### Participants

The sample comprised 519 participants, from three of the eight medical schools in Portugal, namely from the University of Beira Interior (UBI; 203, 39.1 %), the University of Algarve (UAlg; 75, 14.5 %) and the University of Minho (UM; 241, 46.4 %). Most participants (412, 79.4 % of the total) were admitted directly from secondary education through the general admission process into 6-year medical degree programmes (UBI and UM), whereas 107 (20.6 %) were graduates admitted to 4-year graduate entry programmes (UAlg and UM).

Response rates were 50.4 % (UBI), 80.6 % (UAlg) and 82.8 % (UM). The participants ages ranged from 17 to 43 years [M = 21.0, SD = 4.98; M (UBI) = 19.6, SD (UBI) = 2.73; M (UAlg) = 29.4, SD (UAlg) = 4.46; M (UM) = 19.7, SD (UM) = 3.97], of whom 314 were females, (60.5 %; UBI = 56.7 %; UAlg = 54.7 %; UM = 65.6 %). The responding samples from UAlg and UM were similar to their respective school populations for age and sex (*p* < 0.05). The respondents from UBI were slightly younger (19.6 vs. 20.1 years, *p* = 0.005) and slightly less often female (56.7 vs. 67.3 %, *p* = 0.001) than the UBI population. However, the overall respondent sample for the study was similar to the overall medical student population in terms of sex (*p* < 0.001) and age (*p* = 0.574).

### Medical school settings

The types of educational programmes and admission processes of the schools in this study are described below. The University of Algarve offers a graduate entry problem-based learning programme and selects students based on a psychological test and multiple mini interviews (MMIs); University of Minho offers two parallel programmes, one of which is graduate entry (annual intake of 18, representing 15 % of new entrants) selecting students using a science test and MMIs but, similarly to University of Beira Interior, admits most students directly from secondary education through the general application and admissions process, The programmes at the Universities of Minho and Beira Interior are horizontally integrated and are mostly delivered through interactive tutorials, in groups of 30–40 students.

### Instruments

The Big Five personality traits were assessed with the Portuguese version of NEO-Five Factor Inventory (NEO-FFI) with 60 items [18]. It uses a five-point Likert scale ranging from 0 (strongly disagree) to 4 (strongly agree) and can be completed in approximately 15 min. Age, sex and programme type (6 or 4 years/graduate entry programme) were collected in each medical school.

### Procedures and data analysis

Students answered the instruments on paper in two medical schools and online in a computer lab in the other school. Participation was voluntary and individual and data confidentiality was guaranteed. All subjects filled in a consent form after being fully informed of the procedures. Differences on the five dimensions of personality by programme type (graduate entry vs. high school entry; MANOVA 1) and by medical school (MANOVA 2) were evaluated. Two other analysis were performed, considering programme structure and sex as between-subject factors and age as a covariate (MANCOVA 1) and medical school and sex as between-subject factors and age as a covariate (MANCOVA 2). Post-hoc tests (Tukey and Games Howell) were selected depending on the results of the homogeneity of variances (Levene’s test). Results were considered significant for *p* < .05. Data were analyzed with IBM SPSS Statistics v.20.

### Ethics

Research in medical education is exempted from the university’s ethics committee on the ground that this type of research does not aim to answer a research question on health or biomedicine. Nevertheless, this research followed ethical guidelines. Written consent was collected from the participants, prior to the study in accordance with the Declaration of Helsinki ethical principles. Subjects were specifically informed that responses would be kept anonymous, and results would be reported only in aggregate. As all the subjects in the study were adults, there was no need to obtain permission from parents or caretakers. The data collection and database organization were reviewed and authorized by the Portuguese Commission for Data Protection (CNDP:10432/2011). The study obtained retrospective formal approval from our Ethics Review Board prior to publication—Subcomissão de ética para as ciências da vida, process SECVS—071/2013.

## Results

### Exploratory analysis

For each personality dimension, the absolute values of skewness (range between −.589 and .173, for extraversion and neuroticism) and kurtosis (range between −.128 and 1.020 for neuroticism and extraversion) were within the acceptable range of the normal distribution. There were no significant sex differences across medical schools [*χ*_*(2,*=*519)*_^*2*^ = 4.91, *p* = .086, Cramer’s V = .097], but ages differed significantly [*F*_*(2,516)*_ = 232.6, *p* < .001, $\upeta_{\text{p}}^{ 2}$ = 0.474], as students from the University of Algarve were significantly older than those from the Universities of Beira Interior and Minho (*p* < .001). Bivariate correlations showed that age was negatively and significantly correlated with neuroticism (*r* = −.207, *n* = 519, *p* < .001) and positively and significantly correlated with extraversion (*r* = .134, *n* = 519, *p* = .002), openness to experience (*r* = .174, *n* = 519, *p* < .001) and agreeableness (*r* = .147, *n* = 519, *p* = .001). There was no significant correlation between age and conscientiousness (*r* = .069, *n* = 519, *p* = .119).

### Effect of programme structure

MANOVA 1 showed significant differences between programme structure on personality traits *F*_*(5,513)*_ = 7.224, *p* < .001, $\upeta_{\text{p}}^{ 2}$ = 0.07, π = 0.999. Significant effects were found for all the ‘Big Five’ personality traits (Table 1). Graduate entry students presented significantly higher scores than high school entry students on extraversion, openness to experience, agreeableness and conscientiousness but not for neuroticism.

**Table 1:** Means (standard deviations) and MANOVA 1 results for personality traits by programme structure

Personality dimensions	HSE	GE	Total	F_(2,516)_	$\upeta_{\text{p}}^{ 2}$	Π
Neuroticism	21.9 (7.84)	18.5 (6.76)	21.2 (7.75)	17.64***	.021	.853
Extraversion	31.0 (5.68)	32.8 (5.21)	31.4 (5.62)	8.22**	.003	.204
Openness to experience	29.0 (5.41)	31.4 (4.63)	29.5 (5.34)	17.12***	.023	.886
Agreeableness	34.1 (5.28)	36.1 (4.06)	34.5 (5.12)	14.12***	.011	.580
Conscientiousness	34.6 (6.01)	36.3 (5.89)	34.9 (6.02)	6.52*	.009	.459

*HSE* high school entry, *GE* graduate entry

*** *p* < .001; ** *p* < .01; * *p* < .05

### Effects of sex*programme structure controlled for age

**Fig. 1:**
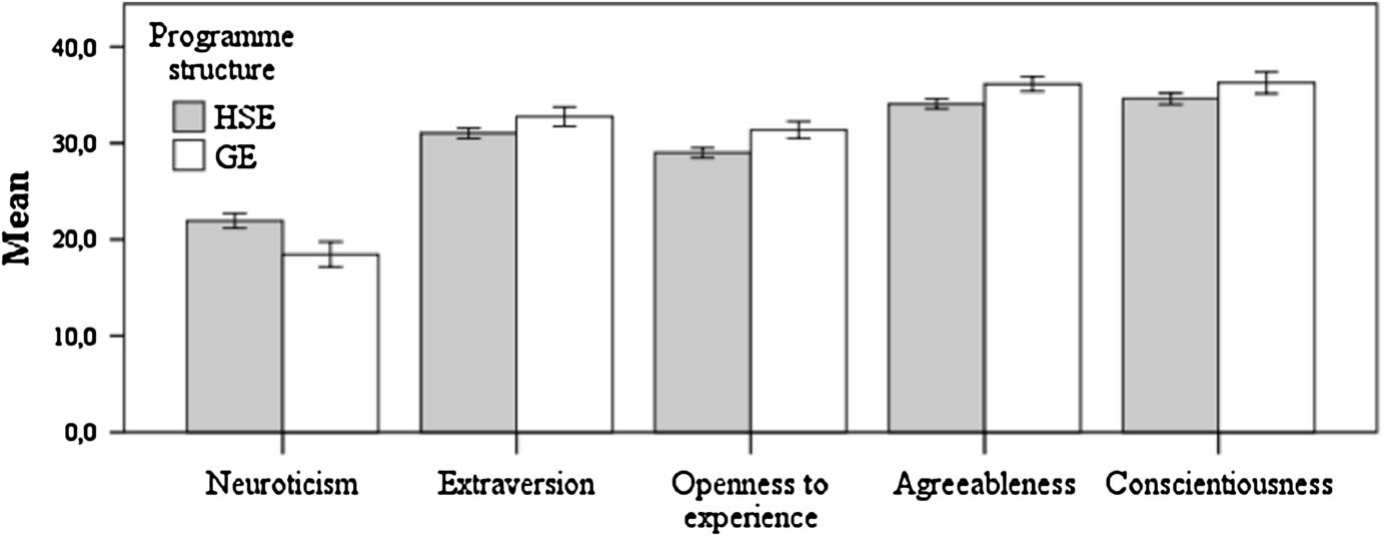
Means and 95 % confidence intervals for personality traits by programme structure

The only significant main effects found for programme structure using sex as between-subject factors and age as a covariate (MANCOVA 1) were sex related [*F*_*(5,510)*_ = 12.021, *p* < .001, $\upeta_{\text{p}}^{ 2}$ = 0.105, π = 1], namely neuroticism [*F*_*(1,514)*_ = 10.607, *p* < .01, $\upeta_{\text{p}}^{ 2}$ = 0.02, π = 0.902], agreeableness [*F*_*(1,514)*_ = 15.784, *p* < .001, $\upeta_{\text{p}}^{ 2}$ = 0.03, π = 0.978] and conscientiousness [*F*_*(1,514)*_ = 20.959, *p* < .001, $\upeta_{\text{p}}^{ 2}$ = 0.039, π = 0.995]. Females presented significantly higher scores. The effect of sex was not significant for extraversion [*F*_*(1,514)*_ < 1] and for openness to experience [*F*_*(1,514)*_ < 1]. The main effect of programme structure [*F*_*(1,510)*_ < 1] and the interaction effect programme structure*sex [*F*_*(1,510)*_ = 1.512, *p* = 0.184, $\upeta_{\text{p}}^{ 2}$ = 0.015, π = 0.532] on personality were not significant. Age was not a significant covariate [*F*_*(1,510)*_ = 1.324, *p* = 0.252, $\upeta_{\text{p}}^{ 2}$ = 0.013, π = 0.471] (Fig. 1).

### Effects of medical school

**Fig. 2:**
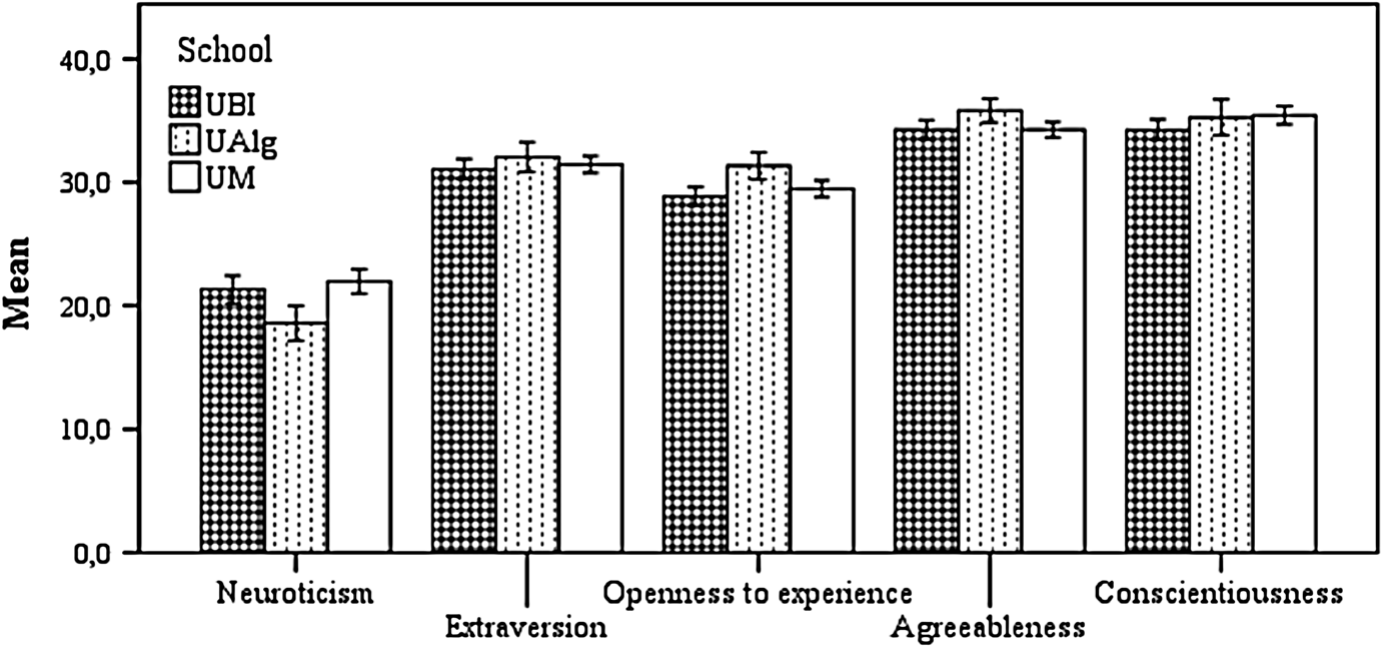
Means and 95 % confidence intervals for personality traits by school

MANOVA 2 showed significant differences between schools on personality traits *F*_*(10,1026)*_ = 3.159, *p* < .001, $\upeta_{\text{p}}^{ 2}$ = 0.03, π = 0.987 (Table 2). Significant effects were found for neuroticism [*F*_*(2,516)*_ = 5.532, *p* < .01, $\upeta_{\text{p}}^{ 2}$ = 0.021, π = 0.853] and for openness to experience [*F*_*(2,516)*_ = 6.089, *p* < .01, $\upeta_{\text{p}}^{ 2}$ = 0.023, π = 0.886]. Neuroticism at the Universities of Beira Interior (M = 21.31, SD = 8.115) and Minho (M = 21.96, SD = 7.751) was significantly higher than at the University of Algarve (M = 18.59, SD = 6.087; all *p* < .05). The openness to experience of students from the University of Algarve (M = 31.33, DP = 4.842) was significantly higher than for students from the Universities of Beira Interior (M = 28.84, SD = 5.414) and Minho (M = 29.46, DP = 5.306). The effect of university on the other personality traits was not significant (Fig. 2).

**Table 2:** Means (standard deviations) and MANOVA results for personalitraits by school

Personality dimensions	UBI	UAlg	UM	Total	F_(2,516)_	$\upeta_{\text{p}}^{ 2}$	π
Neuroticism	21.3 (8.11)	18.6 (6.09)	22 (7.75)	21.2 (7.75)	5.53**	.021	.853
Extraversion	31.1 (6.01)	32.1 (5.26)	31.4 (5.39)	31.4 (5.62)	0.89	.003	.204
Openness to experience	28.8 (5.41)	31.3 (4.84)	29.5 (5.31)	29.5 (5.34)	6.09**	.023	.886
Agreeableness	34.3 (5.6)	35.8 (4.18)	34.3 (4.9)	34.5 (5.12)	2.99	.011	.580
Conscientiousness	34.3 (6.1)	35.3 (6.26)	35.4 (5.83)	34.9 (6.02)	2.26	.009	.459

*UBI* University of Beira Interior, *UAlg* University of Algarve, *UM* University of Minho

*** p < .001; ** p < .01; * p < .05

### Effects of sex*school controlled for age

Introducing sex as a second between-subject factor and age as a covariate (MANCOVA 2) revealed significant results for age [*F*_*(5,508)*_ = 3.656, *p* < .01, $\upeta_{\text{p}}^{ 2}$ = 0.035, π = 0.928] and significant main effects for sex [*F*_*(5,508)*_ = 14.766, *p* < .001, $\upeta_{\text{p}}^{ 2}$ = 0.127, π = 1]. The results were significant for neuroticism [*F*_*(1,512)*_ = 10.718, *p* < .01, $\upeta_{\text{p}}^{ 2}$ = 0.021, π = 0.905], agreeableness [*F*_*(1,512)*_ = 22.045, *p* < .001, $\upeta_{\text{p}}^{ 2}$ = 0.041, π = 0.997] and for conscientiousness [*F*_*(1,512)*_ = 25.86, *p* < .001, $\upeta_{\text{p}}^{ 2}$ = 0.048, π = 0.999] (Table 3).

**Table 3:** Between sex*school comparisons controlled for age

Personality dimensions	UBI		UAlg		UM		Total		F_(1,512)_	$\upeta_{\text{p}}^{ 2}$	π
	M	F	M	F	M	F	M	F			
Neuroticism	19.7	22.5	18.3	18.8	18.9	23.6	19.1	22.6	10.72**	.021	.905
	(8.17)	(7.89)	(6.1)	(6.14)	(7.82)	(7.22)	(7.71)	(7.49)			
Extraversion	31.9	30.4	31.2	32.7	31.7	31.3	31.7	31.2	<1	.000	.050
	(5.71)	(6.18)	(5.17)	(5.31)	(5.56)	(5.31)	(5.54)	(5.68)			
Openness to experience	29.4	28.4	30.8	31.8	29.8	29.3	29.8	29.3	<1	.000	.054
	(5.02)	(5.68)	(4.83)	(4.87)	(5.33)	(5.3)	(5.12)	(5.47)			
Agreeableness	32.8	35.4	34.6	36.9	33	34.9	33.2	35.3	22.05***	.041	.997
	(5.57)	(5.4)	(4.21)	(3.89)	(4.97)	(4.76)	(5.14)	(4.93)			
Conscientiousness	33.5	34.9	32.2	37.8	34.2	36.1	33.6	35.8	25.86***	.048	.999
	(5.88)	(6.23)	(5.73)	(5.57)	(6.64)	(5.27)	(6.18)	(5.74)			

*UBI* University of Beira Interior, *UAlg* University of Algarve, *UM* University of Minho

*** p < .001; ** p < .01

**Fig. 3:**
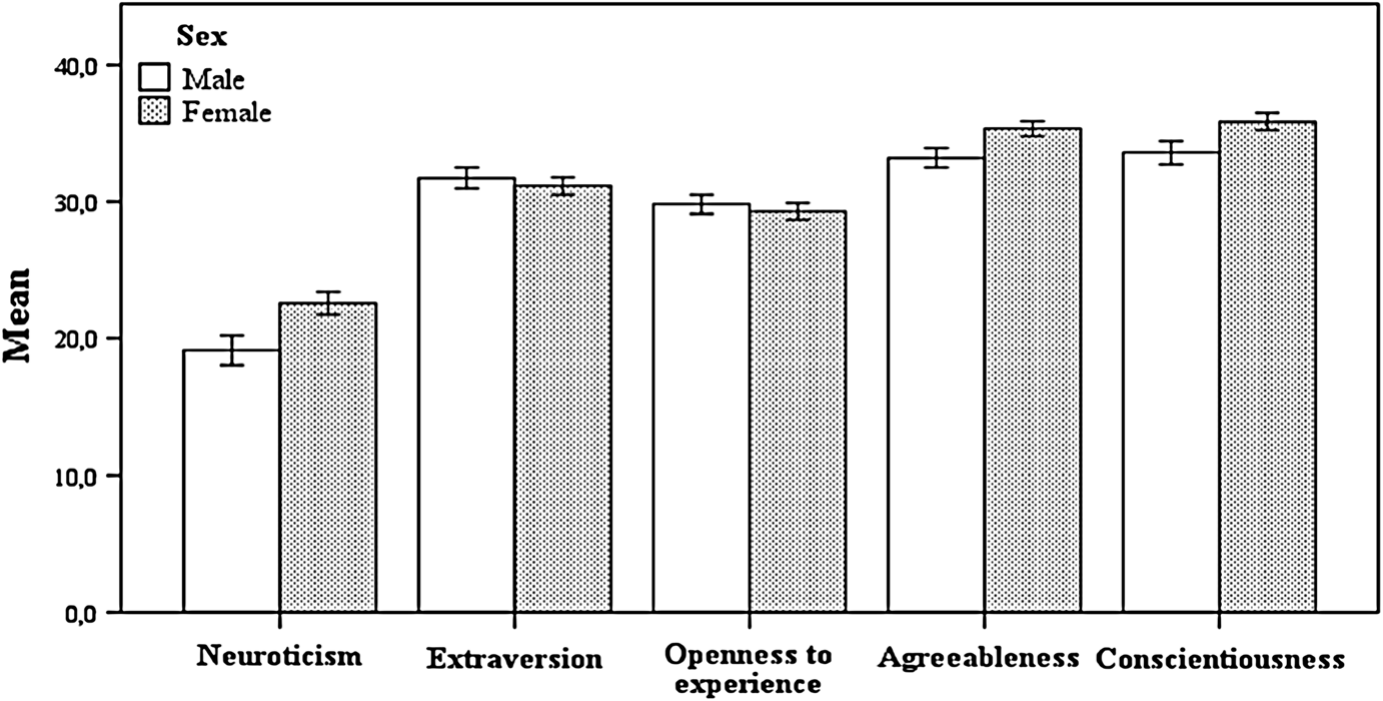
Means and 95 % confidence intervals for personality traits by sex

Female scores were significantly higher for neuroticism (M_M_ = 19.14, SD_M_ = 7.705; M_F_ = 22.57, SD_F_ = 7.486), agreeableness (M_M_ = 33.20, SD_M_ = 5.140; M_F_ = 35.34, SD_F_ = 4.929) and conscientiousness (M_M_ = 35.57, SD_M_ = 6.183; M_F_ = 35.84, SD_F_ = 5.737). There were no significant sex differences on extraversion [*F*_*(1,512)*_ < 1] and openness to experience [*F*_*(1,512)*_ < 1]. The main effect of university [*F*_*(10,1018)*_ = 1.025, *p* = 0.42, $\upeta_{\text{p}}^{ 2}$ = 0.01, π = 0.55] and the interaction effect school*sex [*F*_*(10,1018)*_ = 1.683, *p* = 0.08, $\upeta_{\text{p}}^{ 2}$ = 0.016, π = 0.813] on the ‘Big Five’ traits were not significant (Fig. 3; Table 3).

## Discussion

This exploratory study contributes to the debate on the diversity of student populations in medical schools and shows that graduate entry and high school student populations are different pools of personality profiles [6, 19]. The graduate entry students scored significantly lower on neuroticism and higher on conscientiousness, openness to experience, agreeableness, and extraversion. However, the differentiating element in terms of personality was not related to being or not being a graduate at entry to medical school, but with the fact that graduate students are older students.

The initial analysis showed that, as compared with high school entrants, graduate entry students’ personalities were more tailored for success in medical education. Conscientiousness is the dimension that has been more thoroughly associated with academic success in medical schools [10, 14]. Highly conscientious students are hard-working, persistent and organized [14, 20]. Openness to experience blends openness—which includes traits such as active imagination and aesthetic sensitivity—with intellect—that involves intellectual curiosity and insightfulness. Such sociability related traits have been deemed important for the successful acceptance of the dynamism of clinical settings [10, 20]. Furthermore, one study found that conscientiousness, extraversion and openness were increasingly significant contributors to predict academic success in clinical phases of the medical curriculum [20]. Neuroticism, on the other hand, comprises characteristics such as anxiety, fearfulness, and insecurity in relationships. The lower levels of neuroticism in graduate entry students should reduce the vulnerability of students to stress, both academic and at the workplace [21]. A deeper exploration of the above findings showed that personality differences could be accounted for by age, as significant main effects of the above variables were lost when age was used as a covariate. Controlling for age revealed that the differences that subsisted—neuroticism, agreeableness and conscientiousness—were explained by sex alone. A recent study, conducted in Australia, led to alternative findings, namely that there are small magnitude differences in personality between undergraduate and graduate entry students when age and sex are controlled [17]. Further research must be conducted to clarify these cross-cultural discrepancies.

We further hypothesized that there would be variability of student personality profiles between medical schools and found school effects in the dimensions of neuroticism and openness to experience. However, such differences could not be attributed to medical school or programme structure but were mainly explained by student sex and age. Once more, sex and age emerged as primary factors to explain variability in personality profiles. However, it should be noted that we could be dealing with a selection bias in older graduate entry students since in the programmes of the Universities of Algarve and Minho the students were selected with the use of MMIs. Therefore, within our dataset, the different admissions processes were not important to explain the personality differences. In other words, differences in the personality of graduate entry students in a problem-based learning medical school with a 4-year programme and a high school entry 6-year programme were explained by the age and sex of student populations and not by aspects related to the school. The influence of sex and age is also reported in studies on the general population, in which women are associated with higher scores for neuroticism and agreeableness and lower for extraversion and age correlates with three personality dimensions, negatively for extraversion and openness, and higher on Conscientiousness, a recurrent finding related to the ‘psychosocial maturity’ of the participants [22–24].

Our study suffers from the limitations of being confined to one country, to a limited number of schools and programme types and a possibly biased sample from one of the schools due to lower response rate. We also feel that providing comparative data on academic performance and personality could further enhance our study. We have, however, no such data at this point, which is also a limitation of the present study. In spite of these limitations, it is one of the few multi-institutional studies on differences in medical student personalities. It would be important to confirm our findings, cross-culturally, in schools applying other admission models.

## Conclusion

This study suggests that graduate entry students contribute in desirable ways to the personality pool of medical students and that student age and sex are primary sources of variability. Thus, this study’s major implication for medical education research and practice is that diversifying the personality of admitted students implies diversifying the age and sex of the admitted students.

### Essentials

- We compare personality traits in three different medical schools in Portugal
- We compare personality traits among graduate and school-leaving medical students
- We found differences in all Big Five personality traits
- Personality profiles were different between graduate and undergraduate students
- The differences found were explained by age and gender
